# Synthesis and Thermal Properties of Acrylonitrile/Butyl Acrylate/Fumaronitrile and Acrylonitrile/Ethyl Hexyl Acrylate/Fumaronitrile Terpolymers as a Potential Precursor for Carbon Fiber

**DOI:** 10.3390/ma7096207

**Published:** 2014-09-01

**Authors:** Siti Nurul Ain Md Jamil, Rusli Daik, Ishak Ahmad

**Affiliations:** 1 Chemistry Department, Faculty of Science, Universiti Putra Malaysia, 43400 Serdang, Selangor, Malaysia; E-Mail: nurul_ainjamil@yahoo.com; 2 School of Chemical Sciences and Food Technology, Faculty of Science and Technology, Universiti Kebangsaan Malaysia, 43600 Bangi, Selangor, Malaysia; E-Mail: gading@ukm.edu.my

**Keywords:** redox polymerization, polyacrylonitrile, homopolymer, copolymers, thermal behavior

## Abstract

A synthesis of acrylonitrile (AN)/butyl acrylate (BA)/fumaronitrile (FN) and AN/EHA (ethyl hexyl acrylate)/FN terpolymers was carried out by redox polymerization using sodium bisulfite (SBS) and potassium persulphate (KPS) as initiator at 40 °C. The effect of comonomers, BA and EHA and termonomer, FN on the glass transition temperature (T_g_) and stabilization temperature was studied using Differential Scanning Calorimetry (DSC). The degradation behavior and char yield were obtained by Thermogravimetric Analysis. The conversions of AN, comonomers (BA and EHA) and FN were 55%–71%, 85%–91% and 76%–79%, respectively. It was found that with the same comonomer feed (10%), the T_g_ of AN/EHA copolymer was lower at 63 °C compared to AN/BA copolymer (70 °C). AN/EHA/FN terpolymer also exhibited a lower T_g_ at 63 °C when compared to that of the AN/BA/FN terpolymer (67 °C). By incorporating BA and EHA into a PAN system, the char yield was reduced to ~38.0% compared to that of AN (~47.7%). It was found that FN reduced the initial cyclization temperature of AN/BA/FN and AN/EHA/FN terpolymers to 228 and 221 °C, respectively, in comparison to that of AN/BA and AN/EHA copolymers (~260 °C). In addition, FN reduced the heat liberation per unit time during the stabilization process that consequently reduced the emission of volatile group during this process. As a result, the char yields of AN/BA/FN and AN/EHA/FN terpolymers are higher at ~45.1% and ~43.9%, respectively, as compared to those of AN/BA copolymer (37.1%) and AN/EHA copolymer (38.0%).

## 1. Introduction

Carbon fibers are now important industrially and have gained a wide range of applications, such as in sports utility, military, aerospace industry, automotive, gas adsorption applications, and water treatment. This is due to their superior properties, such as being lightweight, excellent specific strength, stiffness, and excellent thermal, as well as electrical conductivities [1]. Carbon fibers are produced by carbonizing a raw material such as polyacrylonitrile (PAN) fiber. 90% of the carbon fibers produced worldwide are obtained from PAN and the rest are from other raw materials such as phenolic, rayon or pitch fibers [2,3,4]. PAN fibers are used extensively as a source of carbon fibers because their carbon yields are almost twice those of rayon. Hence, PAN fibers have been found to be the most suitable precursors for making high performance carbon fibers [5,6,7,8,9].

The most important process in the manufacturing of carbon fibers from PAN is the cyclization of nitrile groups (during stabilization process) [10] that is influenced by the method of polymerization, nature of comonomers, additives and heat treatment [3,11]. The term stabilization is often used to describe the process of heating the PAN precursor at 200–300 °C under controlled conditions for the succeeding carbonization process and graphitization. Stabilization process is classified as oxidation and cyclization reactions. Oxidation reactions undergo elimination of hydrogen and addition of oxygen. Meanwhile, the cyclization process leads to the formation of a ladder-like structure in the PAN molecule [12,13]. However, the rapid and exothermic process during cyclization causes sudden volatile loss which breaks the chains and damages the fiber structure [14]; consequently, causing mass loss. Too much mass loss due the elimination of HCN or NH_3_ would create uncyclized gaps in the polymer structure which retards the process of cyclization [14] and reduces the formation of a ladder-like structure in polymer. This is unfavorable to the mechanical strength of carbon fiber. Hence, the objectives of this research were to reduce the temperature and heat evolution during the stabilization process.

On the other hand, PAN has a high melting point (~320 °C), which is attributed to chain stiffness and nitrile dipolar interaction between polymer chains [15]. However, the melting endotherm of PAN cannot be observed because PAN undergoes nitrile cyclization at temperatures between 180 and 220 °C, followed by degradation at a higher temperature [16] before it melts [12]. Therefore, the PAN precursor commonly undergoes solution-spun which requires solvent recovery; hence, giving higher processing costs [12]. In order to gain cost effectiveness in producing carbon fiber, melt spinning processing is a good option to replace solution spinning. It is important to reduce the T_m_ of PAN to permit melt processing. Previous, melt processability of PAN precursor was studied by incorporating PAN with comonomers, which act as plasticizer: 1-butyl-3-methylimidazolium chloride [17], vinylimidazole [12], methacrylic acid [18], methyl acrylate [19], and itaconic acid [20]. However the thermal stabilization properties have not been discussed in details.

The high chain stiffness contributed to the high T_m_ value of polymer. The chain mobility (that can be determined by glass transition temperature, T_g_) of PAN was also influenced by the chain stiffness attributed to the dipolar interaction between nitrile groups. Thus, a high T_g_ indirectly indicates that PAN has a high T_m_ that prevents PAN from undergoing melt processing [21]. Hence, to investigate the melt processing potential of PAN, T_g_ of polymers was searched for to reflect the T_m_ of polymers. Therefore, in this present work, two acrylate comonomers with different molecular size namely butyl acrylate (BA) and ethyl hexyl acrylate (EHA), were incorporated to disrupt the order of polymer chains in the PAN system. Acrylate comonomer acts as defects and helps to reduce the dipole-dipole interactions and long-range order present in the PAN system [22], thereby, reducing its glass transition temperature and lowering its processing temperature [23]. This will enable the melt processing of terpolymer.

Although acrylate comonomer is able to reduce the T_g_ [24], it has been reported that acrylate comonomer would result in copolymer with low char yield [22,25]. To overcome the stabilization temperature problem and the low char yield, the acidic comonomers that are commonly used [11,26,27] to facilitate the stabilization process can be replaced by fumaronitrile (FN) as a termonomer. We previously reported that the incorporation of FN slightly reduced the initial exotherm temperature during the stabilization process with a slower rate of nitrile cyclization. Hence, it significantly increased the char yield of PAN [28]. It is anticipated that the presence of two nitrile groups in FN is favorable in reducing the weight loss during stabilization process; hence, increasing the char yield during carbonization [28]. A high amount of char yield indicates a high amount of carbon content in fibers which leads to carbon fibers having good mechanical properties [22,25].

**Figure 1:**
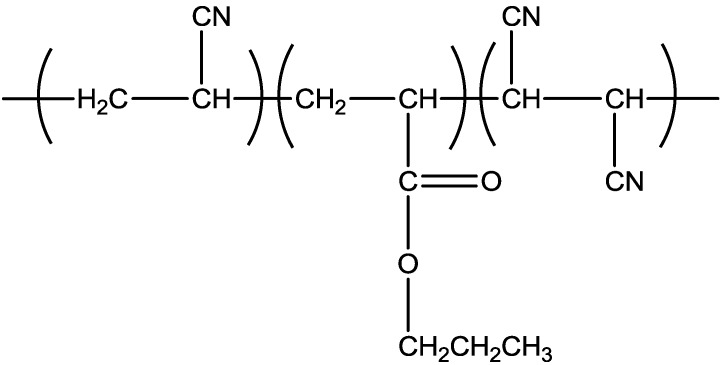
Poly(acrylonitrile/butyl acrylate/fumaronitrile).

**Figure 2:**
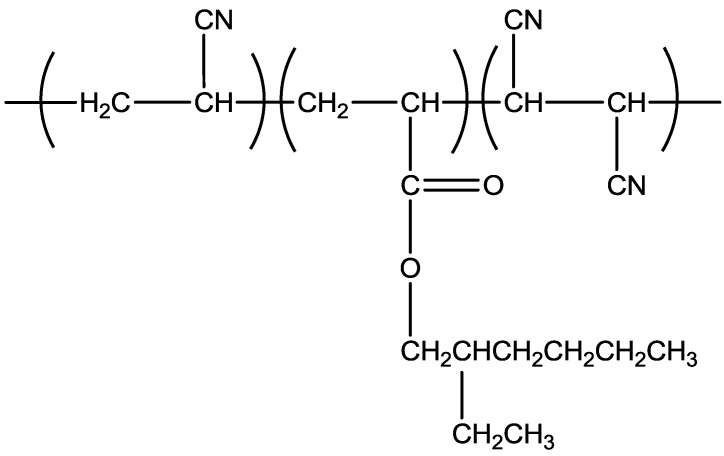
Poly(acrylonitrile/ethyl hexyl acrylate/fumaronitrile).

Overall, this study focused on producing PAN terpolymers with low T_g_, which results in better stabilization process by incorporating acrylate comonomers into the PAN system. Figure 1 and Figure 2 show the formation of poly(acrylonitrile/butyl acrylate/fumaronitrile) and poly(acrylonitrile/ethyl hexyl acrylate/fumaronitrile), respectively.

## 2. Results and Discussion

### 2.1. FTIR Spectroscopy

**Figure 3:**
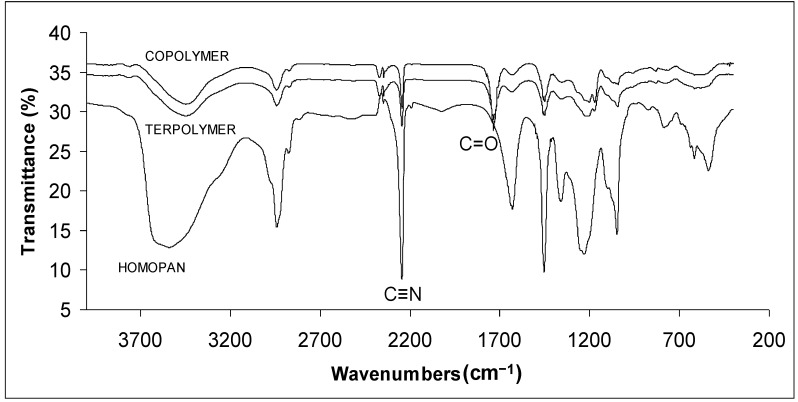
FTIR spectra of PAN homopolymer, copolymer, and terpolymer.

The IR spectra of PAN, its copolymer and terpolymer are shown in Figure 3. In all cases, the bands in the region of 2943 cm^−1^ were assigned to C–H stretching in CH, CH_2_ and CH_3_. The bands at 1450 cm^−1^, 1353 cm^−1^ and 1204–1199 cm^−1^ were due to the C-H vibrations of different modes. The band at 2244 cm^−1^ indicated the absorption of nitrile groups in PAN homopolymer, copolymers and terpolymers [26]. The band in the region of 1632 cm^−1^ was assigned to the stretching of NH_2_ groups due to acrylamide formation by partial hydrolysis of acrylonitrile units during the polymerization process using redox initiator [27]. The strong band in the range of 1735 cm^−1^ in copolymer and terpolymer spectra was due to the C=O stretching [26,29]. The disappearance of bands at 2238–2239 cm^−1^ that are due to the stretching of unsaturated nitriles of FN [30] confirms that FN was incorporated into terpolymers. It was reported that fumaronitrile cannot homopolymerize but copolymerizes easily under free radical condition [31].

### 2.2. Conversions and Composition Analysis

Table 1 shows the conversions of polymerization for PAN homopolymer, copolymers and terpolymers. The conversions of AN/BA copolymer and AN/EHA copolymers are 75% and 77%, respectively, which are lower compared to the PAN homopolymer (85%), as expected. Similarly, AN/BA/FN and AN/EHA/FN terpolymers were also lower (61%–77%) compared to the PAN homopolymer. This is probably due to the absence of abnormalities or defects in the PAN homopolymer chains since polymerization was not incorporated with other monomers. In addition, the method used to obtain PAN homopolymer by redox method as reported before [23] was the most suitable and under optimum conditions. Besides, redox polymerization that was carried out under mild condition (lower energy of activation) lowered the possibility of side chain reactions; hence, giving high yield for the PAN homopolymer [32].

**Table 1:** Composition of reacted monomers in polyacrylonitrile (PAN) homopolymer, copolymer and terpolymer.

Monomers feed (mol%), M AN/comonomer/FN	Monomers conversion (%)	Monomers composition (mol%), m AN/comonomer/FN	Reacted monomer (%) m/M × 100 AN/comonomer/FN	Actual composition (mol%) AN/comonomer/FN
100/0/0	85	85/0/0	85/0/0	100/0/0
**AN/BA/FN**				
95/5/0	77	71.35/4.50/0	75/90/0	94.1/5.9/0
90/10/0	75	64.06/8.90/0	71/89/0	87.8/12.2/0
90/2/8	73	64.58/1.78/6.24	72/89/78	88.9/2.5/8.6
90/4/6	67	57.29/3.48/4.74	64/87/79	87.5/5.3/7.2
90/6/4	68	58.33/5.10/3.08	65/85/77	87.7/7.7/4.6
90/8/2	61	49.48/6.96/1.51	55/87/76	85.4/12/2.6
**AN/EHA/FN**				
95/5/0	76	69.79/4.55/0	73/91/0	93.9/6.1/0
90/10/0	77	64.58/9.0/0	72/90/0	87.8/12.2/0
90/2/8	71	61.46/1.82/6.32	68/91/79	88.3/2.6/9.1
90/4/6	68	57.29/3.60/4.62	64/90/77	87.45/5.5/7.1
90/6/4	67	55.21/5.28/3.12	61/88/78	86.8/8.3/4.9
90/8/2	66	53.13/6.96/1.54	59/87/77	86.2/11.3/2.5

The composition of reacted comonomer and termonomer in the polymer increased as the amount of comonomer and temonomer was increased in the feed (Table 1). The composition of BA and EHA comonomers in copolymer system is higher (~90%) when compared to that of AN (~70%). This is due to the ester monomers that are more reactive in radical copolymerization, as reported by other researchers [33].

In terpolymer system, the composition of comonomers (BA and EHA) is higher (85%–91%) as compared to that of AN (55%–72%) and FN (76%–79%). The rate of copolymerization is greatly affected by the concentration and polarity of monomers [16]. During the initial redox polymerization, propagation occurred in an aqueous phase which also contains the reaction initiating species. Due to higher solubility of AN and FN, these two monomers have a higher probability to combine with initiating species (SBS and KPS) at this initial homogenous stage [29]. When the propagating chains grow big enough, they will be precipitated and polymerization takes place both in water and on the surface of the precipitated particles, which means that the polymerization has changed from a homogenous to heterogeneous reaction [34]. At this stage, AN and FN which are more hydrophilic, remain in the aqueous phase and are now in contact with the polymerizable polymer chains [34]. Meanwhile, BA and EHA comonomers which are more hydrophobic become buried in the particle core of oligomers and continuously propagate to form polymer [29]. Due to better chances of BA and EHA to polymerize during the heterogeneous stage, the reaction or conversion of BA and EHA comonomers is the highest compared to AN and FN, respectively.

### 2.3. DSC Studies

#### 2.3.1. Effect of Comonomer on T_g_

As shown in Table 2, the PAN homopolymer showed the highest T_g_ at 210 °C. This can be explained by the nitrile-nitrile dipolar interactions that provide regularity sequence along the PAN homopolymer chains, resulting in retardation of the chains movements. However, the introduction of 10% BA and 10% EHA comonomers greatly lowered the glass transition (T_g_) of AN/BA copolymers to 70 °C and 63 °C, respectively, compared to PAN homopolymer (210 °C). Since the acrylate groups affect the regularity sequence along the PAN homopolymer chains, they reduce the dipolar interaction of PAN chains. Less dipolar interactions increase the free volume of the PAN system, thus enhance the mobility of polymer chains [24,35] and thereby depress the T_g_. With the same amount of comonomers feed (10%), the T_g_ of AN/EHA copolymer was lower at 63 °C compared to the AN/BA copolymer (70 °C). This is due to the larger size of acrylates which provide larger interruptions to the regularity sequence of the PAN system and facilitate the chain mobility by reducing the nitrile-nitrile dipolar interactions along the polymer chains. On the other hand, it was shown that AN/EHA/FN 90/2/8 terpolymer gave similar T_g_ value with the AN/BA/FN 90/4/6 terpolymer at 67 °C. This indicates that a lower amount of bulkier acrylate comonomer (EHA) provides a comparable T_g_ to a smaller size of acrylate group (BA). Incorporating the least amount of acrylates is favorable to minimize the interruption along the PAN homopolymer chains. High interruption on the nitrile sequence along PAN chains leads to the low formation of char yield after thermal stabilization [22].

Low T_g_ is favorable in this study because it reflects low T_m_ that lowers its processing temperature, thereby facilitating the melt extrusion/spinning of PAN homopolymer [12,22]. A PAN system with a potential to undergo melt spinning is desirable to avoid high expenses on solvent recovery and recycling.

In the AN/BA/FN and AN/EHA/FN terpolymers obtained, it was seen that as the amount of FN increased from 2 to 8 mol%, the T_g_ of the PAN system slightly increased. This could be attributed to some potential interactions between the nitrile groups of FN and AN, which could reduce the chain mobility and lead to a higher T_g_.

**Table 2:** Glass transition temperature of PAN homopolymer, copolymers, and terpolymers.

AN/comonomer/FN (mol%)	Glass transition, T_g_ (°C)
100/0/0	210 ± 1
**AN/BA/FN**	
90/2/8	69 ± 1
90/4/6	67 ± 2
90/6/4	67 ± 2
90/8/2	63 ± 1
90/10/0	70 ± 1
**AN/EHA/FN**	
90/2/8	67 ± 1
90/4/6	65 ± 1
90/6/4	63 ± 1
90/8/2	60 ± 2
90/10/0	63 ± 1

#### 2.3.2. Effect of Comonomer and Termonomer on Stabilization Temperature

FN initiates the cyclization of the nitrile group at lower temperature as shown in Table 3. The T_i_ (initial cyclization temperature) for AN/BA/FN terpolymers is about 228 °C which is lower as compared to that of AN/BA copolymer (264 °C). Similar observation was found in the case of AN/EHA/FN terpolymers in the sense that T_i_ was lower (220 °C) as compared to that of the AN/EHA copolymer (263 °C). This behavior could be attributed to the interruption of polymer chain sequence provided by the FN group.

**Table 3:** DSC data of PAN homopolymer, copolymers and terpolymers.

AN/comonomer/FN (mol%)	T_i_ (°C)	T_f_ (°C)	∆T (°C)	∆H (J·g^−1^)	∆H/∆t (J·g^−1^·min^−1^)
100/0/0	246	345	99	758	77
**AN/BA/FN**					
90/10/0	264	321	57	590	104
90/2/8	228	309	81	371	46
90/4/6	233	317	84	392	47
90/6/4	241	331	90	437	49
90/8/2	260	353	93	489	53
**AN/EHA/FN**					
90/10/0	263	306	43	523	122
90/2/8	250	321	71	321	45
90/4/6	221	291	70	345	49
90/6/4	220	304	84	356	42
90/8/2	241	327	86	381	44

Since FN has two nitrile groups on its molecule, some intra or intermolecular interactions occurred between FN, acrylonitrile and acrylates which ease the initial cyclization process [28].

**Figure 4:**
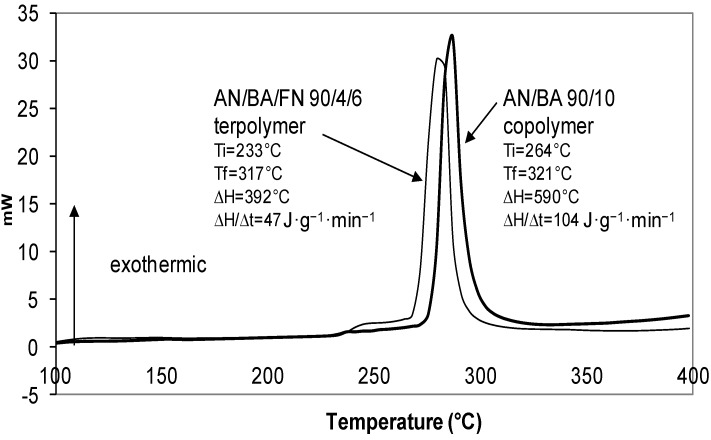
DSC thermograms of AN/BA 90/10 copolymer and AN/BA/FN 90/4/6 terpolymer.

**Figure 5:**
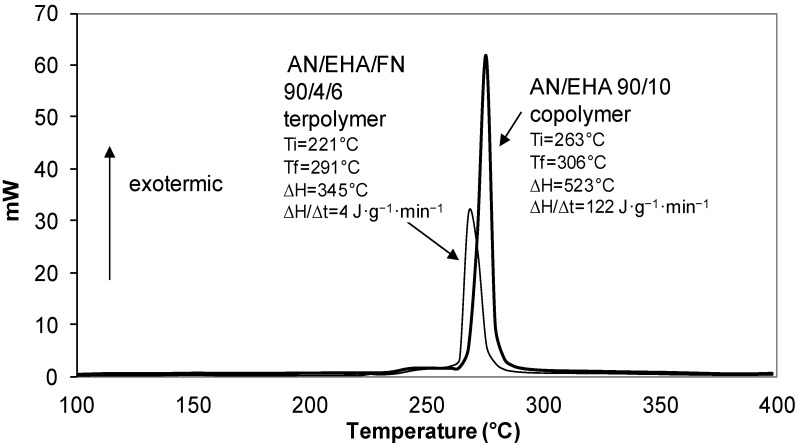
DSC thermograms of AN/EHA 90/10 copolymer and AN/EHA/FN 90/4/6 terpolymer.

The DSC thermogram that demonstrated the cyclization behavior of AN/BA/FN 90/4/6 terpolymer is shown in Figure 4. It reveals that AN/BA/FN terpolymer has a broader exothermic peak, as indicated by the greater value of ∆T (T_f_ − T_i_) in Table 3. Similarly, the ∆T of the AN/EHA copolymer was lower (43 °C) as compared to the value of AN/EHA/FN terpolymer (∆T = 70 °C). A broader peak suggests that terpolymer has a slower propagation reaction for producing a ladder-like polymer [10,36], whereas the peaks observed for the AN/BA (Figure 4) and AN/EHA copolymer (Figure 5) were more intense. On the other hand, the total heat of exothermic reaction, ∆H in the case of terpolymers is lower as compared to that of copolymers. This observation suggests a different reaction mechanism that might have taken place during stabilization with a relatively much slower propagation [11] as a result of incorporation of FN.

For instance, the ∆H/∆t for AN/BA 90/10 copolymer and AN/EHA 90/10 copolymer are 104 J·g^−1^·min^−1^ and 122 J·g^−1^·min^−1^ respectively, which are higher than that of AN/BA/FN 90/4/6 terpolymer (47 J·g^−1^·min^−1^) and AN/EHA/FN 90/4/6 terpolymer (49 J·g^−1^·min^−1^). This shows that the exothermic reaction in the case of terpolymers takes a much longer time to be completed with a lower heat liberation per time. This leads to a better heat distribution during stabilization process, thereby increasing the formation of a ladder-like structure that leads to a better quality of carbon fiber [11]. 

### 2.4. TGA Studies

The TGA was carried out to obtain the thermal stability and get an estimate of the carbon yield. As the samples are subjected to heating, it starts to actively cyclize at temperatures greater than 220 °C, leading to char formation at the end of heat treatment. The results give a preliminary estimate of the carbon yield of the polymers [4,22]. As shown in Table 4, the char yield of PAN is low at 47.7% because the heat dissipation is more difficult in the PAN homopolymer due to its poor conduction of heat [22]. Hence, excessive localized heating can lead to chain scission and subsequently lowers the char yield. On the other hand, as the amount of comonomers (BA and EHA) increased from 2 mol% to 10 mol%, the char yields of AN/BA/FN and AN/EHA/FN terpolymers were significantly reduced to 38.0% and 37.1%, respectively. This is due to the acrylate comonomer units that act as defects in the PAN chains to reduce the formation of a ladder-like structure along the polymer chains, resulting in a lower char yield [22]. However, by incorporating 2–6 mol% of FN in the case of AN/BA/FN and AN/EHA/FN terpolymers, the char yield of terpolymers increases to 45.1% and 43.9%, respectively. Some interactions between FN units that have two nitrile groups each and AN system ease the stabilization process. This minimizes the chain scission reactions and the loss of volatile products, leading to lesser weight loss.

**Table 4:** Char yields of PAN homopolymer, copolymers and terpolymers.

AN/comonomer/FN (mol%)	Weight loss (%)				Char yield (%)
	Step 1 30–250 °C	Step 2 Part 1 250–350 °C	Step 2 Part 2 350–600 °C	Step 3 600–950 °C	
100/0/0	1.61	23.21	13.17	14.27	47.7
**AN/BA/FN**					
90/10/0	0.18	34.62	20.14	7.06	38.0
90/2/8	0.15	29.99	14.69	10.04	45.1
90/4/6	0.18	32.52	15.01	8.43	43.9
90/6/4	0.17	34.21	15.98	9.53	40.1
90/8/2	0.19	33.76	17.01	9.06	40.0
**AN/EHA/FN**					
90/10/0	0.45	35.80	15.0	11.67	37.1
90/2/8	1.67	30.18	14.14	10.12	43.9
90/4/6	0.24	33.21	15.73	8.34	42.5
90/6/4	0.78	35.01	16.23	7.79	40.2
90/8/2	1.12	38.65	14.29	6.84	39.1

**Figure 6:**
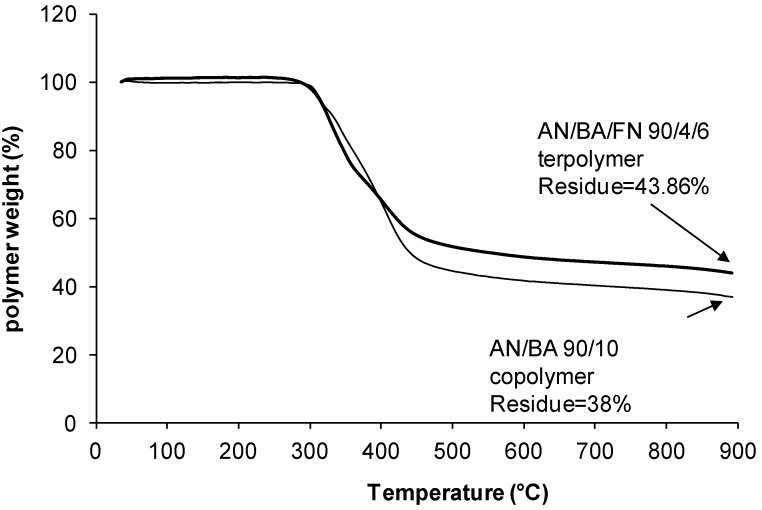
TGA thermograms of AN/BA 90/10 copolymer and AN/BA/FN 90/4/6 terpolymer.

**Figure 7:**
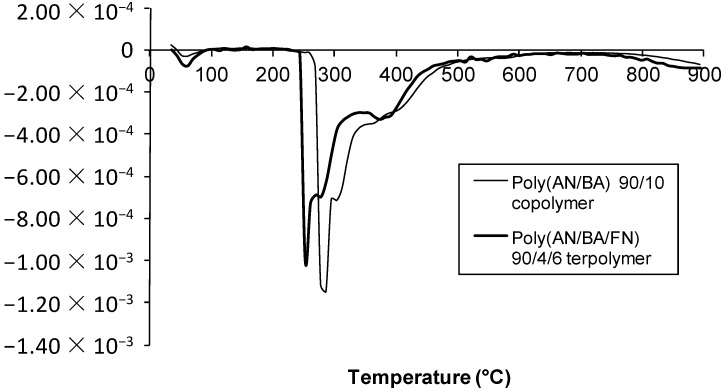
DTG curves of AN/BA 90/10 copolymer and AN/BA/FN 90/4/6 terpolymer.

**Figure 8:**
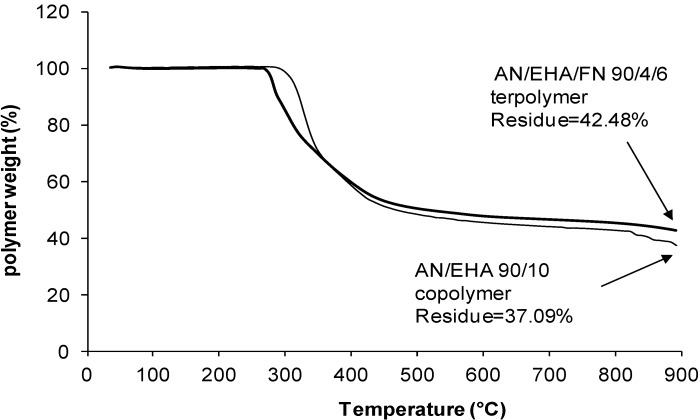
TGA thermograms of AN/EHA 90/10 copolymer and AN/EHA/FN 90/4/6 terpolymer.

**Figure 9:**
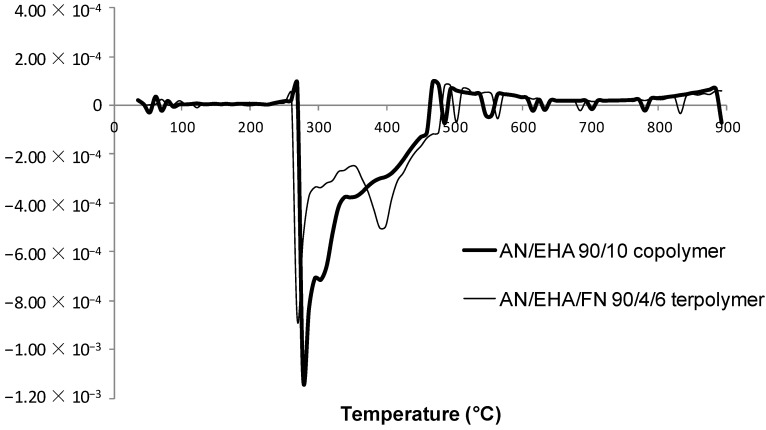
DTG curves of AN/EHA 90/10 copolymer and AN/EHA/FN 90/4/6 terpolymer.

The degradation behavior of the AN/BA copolymer and its terpolymer as well as AN/EHA copolymer and its terpolymer is shown in Figure 6, Figure 7, Figure 8 and Figure 9. The TGA thermograms of copolymer and its terpolymer (Figure 6 and Figure 8) showed a three-step weight loss. As shown in Figure 7 and Figure 9, and listed in Table 4, the weight loss in the region of 30–100 °C is the vaporization of moisture. The first step is up to 250 °C, where the weight loss is not substantial. The degradation in this region is due to the evolution of HCN and NH_3_ [11]. The second step is between 250 and 600 °C with a very rapid weight loss. This is due to the cyclization and oxidation reactions which are exothermic and contribute to the formation of ladder polymer structure in the polyacrylonitrile molecule [13]. The third step starts at 600 up to 950 °C with a quite steady weight loss. In the case of AN/BA copolymer, the DTG curve (Figure 7) shows that the weight loss in the first step is 0.18%, while in the second step, starting from 250 to 600 °C, the weight loss is very high at 54.76% and appears as a broad DTG curve. The final step shows a steady and slow weight loss (7.06%) up to 950 °C. Overall, the total char yield is 38.0% as shown in Figure 6. Meanwhile, the AN/EHA copolymer showed a similar trend with the highest weight loss at 50.80% in the second step. Note that in Table 4 with the same feed amount of acrylate comonomer, the weight loss in the second step (part 1; 250–350 °C) for AN/EHA copolymer was slightly higher (35.80%) compared to that of the AN/BA copolymer (34.62%). This is probably due to the size of EHA comonomer which is bulkier compared to the BA; therefore, it provides more defects in the polymer system and increases the weight loss during degradation in the second step (part 1) that refers to the exothermic process during stabilization. As a result, the char yield of AN/EHA copolymer was slightly lower at 42.5% compared to that of the AN/BA copolymer (43.9%). However, as shown in Table 4, the AN/EHA/FN 90/2/8 obtained a comparable value of char yield with AN/BA/FN 90/4/6 (43.9%). This observation shows that the amount and size of acrylate group affect the degradation behavior of the PAN system. It is important to minimize the amount of acrylate group to reduce the weight loss during heat treatment of the PAN system.

As shown in Table 4, the final char yields of AN/BA copolymer and AN/EHA copolymer are 38.0% and 37.1%, respectively, which are lower than that of PAN (47.7%). This is expected to be due to the effect of acrylate group that is anticipated to interrupt the nitrile sequence along the polymer chains, resulting in high weight loss after stabilization up to 950 °C. It should be noted that the role of acrylate comonomer as diluent is to enhance the chain mobility of the PAN system and, hence, reduces the T_g_ value.

On the other hand, for the AN/BA/FN 90/4/6 terpolymer (Figure 7), the weight loss in the first step is not substantial at only about 0.18%. The weight loss of the second step is 47.53%. The DTG curve shows that the degradation occurred at a faster rate as shown by the more intense but a smaller peak as compared to that of the AN/BA copolymer. Whereas, in the third step, the weight loss is about 8.43% and results in a total char yield of 43.9%, which is higher compared to AN/BA copolymer (38.0%). Likewise, the thermogram of AN/EHA/FN terpolymer (Figure 9) exhibited a smaller peak during degradation in the second step in comparison to that of the AN/EHA copolymer. Thus, AN/EHA/FN terpolymer gave a higher char yield (42.5%) than that of the AN/EHA copolymer (37.1%).

This result indicates that FN successfully facilitates the stabilization process and improves the thermal stability of AN/acrylate/FN. While one of the nitrile groups involved in the fragmentation of the chain, another nitrile group undergoes cyclization process to form a ladder-like structure as stabilized fibers. This shows that the incorporation of FN into terpolymer increases the possibility to form a ladder-like structure during stabilization. A perfect formation of a ladder-like structure leads to a high char yield during carbonization of AN/acrylate/FN terpolymer. Char yield is very important because it provides a realistic indication of the final carbon content of carbon fiber [22].

## 3. Experimental Section

### 3.1. Synthesis and Characterization

Polymerization of acrylonitrile (AN) with butyl acrylate (BA) and ethyl hexyl acrylate (EHA) as comonomers and fumaronitrile (FN) as termonomer was prepared by redox method. Sodium bisulfite (SBS) and potassium persulfate (KPS) were used to initiate the polymerization. The synthesis of poly (AN/BA/FN) 90/4/6 (mole ratios) was carried out in a three-necked flask at 40 °C under nitrogen atmosphere. The flask was fitted with a condenser and the third neck was used for nitrogen purging. Deionized water (200 mL) was added into the flask, and the temperature was increased to 40 °C. After 30 min, AN monomer (172.8 mmol) was added into the reaction mixture followed by BA comonomer (7.7 mmol) and FN termonomer (11.5 mmol). Finally, SBS (1 mmol) and KPS (1 mmol) were added as redox initiators. The mixture was then stirred under nitrogen at 40 °C, and the polymerization was allowed to proceed for 3 h. The polymer formed was precipitated, filtered, washed successively with methanol and deionized water and dried under vacuum at 45 °C till a constant weight was obtained [23,28]. Polyacrylonitrile homopolymer and copolymer were also prepared in the same way.

FTIR spectra of PAN homopolymer, copolymers and terpolymers were recorded on a Perkin Elmer GX infrared spectrophotometer using KBr pellets.

### 3.2. Composition Analysis of Polymers

The composition of reacted monomers in copolymers and terpolymers was determined from residual monomer concentration data. The residual monomer concentrations of withdrawn samples were obtained using a Gas Chromatography system. The polymers obtained were firstly mixed with a defined amount of methanol to precipitate and isolate the polymer from the reaction medium (water). The residual monomer remained in water. A defined portion of supernatant was injected for GC analysis [37].

**Figure 10:**
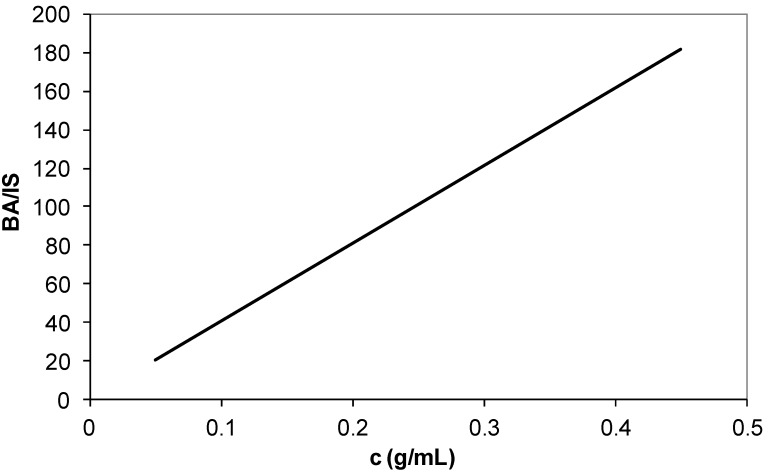
Calibration curve of butyl acrylate (BA) comonomer. IS: Internal standard.

**Figure 11:**
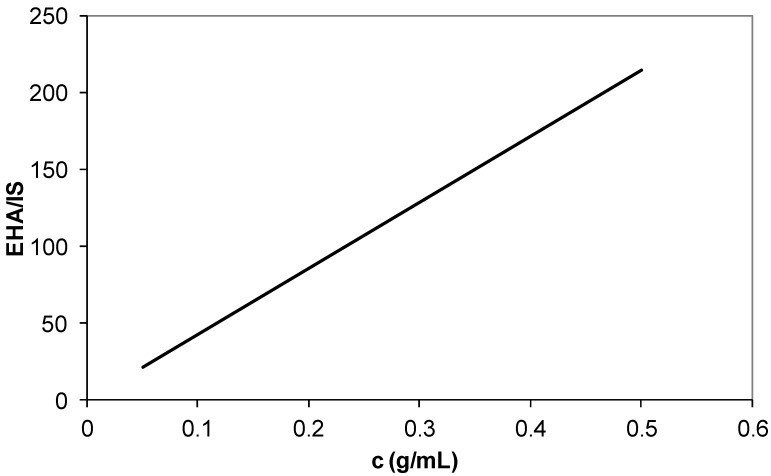
Calibration curve of ethyl hexyl acrylate (EHA) comonomer. IS: Internal standard.

**Figure 12:**
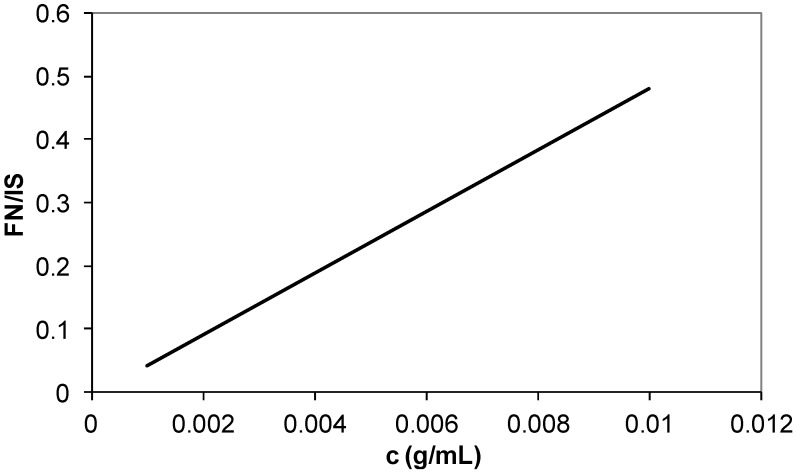
Calibration curve of fumaronitrile (FN) termonomer. IS: Internal standard.

The calibration curves of butyl acrylate (Figure 10), ethyl hexyl acrylate (Figure 11) and fumaronitrile (Figure 12) were obtained by using GC system. Undecane was used as an internal standard for calibration curve and sample analysis. The peak area served as calibration parameter. The composition of reacted acrylonitrile in copolymers and terpolymers was calculated using Equation (1):

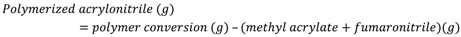
(1)

### 3.3. Differential Scanning Calorimetry (DSC)

Samples in powder form (~5) mg were used for DSC analysis. The glass transition temperature (T_g_) of the samples was determined using a Mettler-Toledo Differential Scanning Calorimeter, Mettler Toledo International Inc., Greifensee, Switzerland. The heating rate administered was 10 °C·min^−^^1^ and the samples were heated from room temperature to 200 °C, cooled back to room temperature at the same rate, and reheated to 200 °C. The stabilization temperature was obtained by heating the samples from room temperature to 400 °C at 10 °C·min^−^^1^.

### 3.4. Thermogravimetric Analysis

Thermogravimetric analysis was carried out using a Mettler-Toledo TGA instrument, Mettler Toledo International Inc. in nitrogen from room temperature to 950 °C at a heating rate of 10 °C·min^−^^1^. A sample size of ~15 mg in the form of fine powder was used. To establish the relationship between the weight loss and temperature, TG thermograms and DTG curves were recorded.

## 4. Conclusions

AN/BA copolymer, AN/BA/FN terpolymer, AN/EHA copolymer and AN/EHA/FN terpolymer were successfully synthesized by redox method with high conversion of monomers. BA and EHA comonomers greatly reduced the T_g_ of their copolymers respectively, and this indirectly indicates that acrylate group enhanced the mobility of the polymer chains and facilitated their flowability. Thus, AN/BA and AN/EHA copolymers have a potential to undergo melt processing. Incorporating a lower amount of a bulkier acrylate comonomer (EHA) results in similar T_g_ to a higher amount of smaller acrylate comonomer (BA). This observation shows that the size of acrylate group affects the chain mobility and the defects on the PAN chains can be minimized by incorporating a bulkier acrylate group for further heat treatment to obtain char yield.

Incorporating BA and EHA resulted in a lower char yield as compared to the PAN homopolymer. FN termonomer incorporated into the PAN system has been shown to facilitate the stabilization process to occur at a lower temperature with lower heat liberation. Therefore, the char yield of AN/BA/FN terpolymer is higher (45.1%) than that of AN/BA copolymer (38.0%). Similarly, the AN/EHA/FN terpolymer also showed higher char yield at 43.9% compared to the AN/EHA copolymer at 37.1%. This research suggests that the AN/BA/FN and AN/EHA/FN terpolymers system has a potential to undergo melt spinning and has a good thermal stability due to a higher char yield which reflects higher carbon content after the heat treatment. The amount of the acrylate group can be minimized to reduce the defect in the PAN system by incorporating a bulkier acrylate group.
